# Childhood Asthma and Smoking: Moderating Effect of Preterm Status and Birth Weight

**DOI:** 10.7759/cureus.14536

**Published:** 2021-04-17

**Authors:** Chukwuemeka E Ogbu, Stella C Ogbu, Dibya Khadka, Russell S Kirby

**Affiliations:** 1 College of Public Health, University of South Florida, Tampa, USA; 2 Department of Biology, East Tennessee State University, Johnson City, USA; 3 Department of Biology, University of North Texas Denton, Denton, USA

**Keywords:** prematurity, parental smoking, asthma, second-hand smoke, effect modification, birth weight

## Abstract

Introduction

Although studies have examined the association between childhood asthma and parental smoking and secondhand smoke, little research has explored the moderating role of birth weight and prematurity (BWP) status on this association. We examined the association between secondhand smoke exposure, asthma, and asthma severity in children aged six to 17 as well as the modifying effect of BWP on parental smoking and asthma.

Methods

We used data from 36,954 children from the National Survey of Children’s Health 2017-2018. In addition to univariate analysis, adjusted and unadjusted logistic regression models were used to estimate the effect of secondhand smoke on asthma. The interaction term between parental smoking and BWP was tested. Multinomial regression was used to evaluate the association between secondhand smoke on asthma severity.

Results

About 15.1 % of children had asthma and 15.4% of parents reported smoking. Odds of asthma were higher in children living with an outdoor (AOR, 1.27; 95% CI, 1.06-1.52) and indoor (AOR, 1.46; 95% CI, 1.01-2.11) smoker in the adjusted model. The association of parental smoking with asthma differed by birth weight and premature status. Normal weight children who are premature had the highest odds ratio (AOR, 2.15; 95% CI, 1.2-3.86). In the multinomial model, low birth weight and premature children had higher odds of mild (AOR, 1.90; 95% CI, 1.40-2.56) and moderate/severe (AOR, 1.81; 95% CI, 1.16-2.84) asthma compared to the no asthma group.

Conclusion

The Association of parental smoking on asthma was modified by BWP. Focused asthma interventions in children should inquire about BWP status as well as parental smoking and household smoke exposure to reduce asthma morbidity and mortality.

## Introduction

Asthma is a common chronic inflammatory airway disease and a leading chronic disease among children aged 0-17 years, and it is more common in children than in adults [1]. The prevalence rates of asthma decreased from 9.5% in 2016 to 7.5% in 2018 and childhood asthma declined 12.4% in Age-Standardized Years Lived with Disability rates, indicating a decrease in disease burden in U.S. children [1-2]. However, the chronic nature of this illness, resultant complications of this disease if not appropriately managed, and the association of asthma with impaired physical, emotional, cognitive, and social functioning of the child impairs the quality of life of in children and warrants attention [3]. Moreover, asthma is predicted to continue to be one of the leading causes of childhood respiratory illnesses and increased disease burden in the future [4]. Therefore, preventing asthma is an important public health objective.

Childhood smoke exposure is an important risk factor for asthma development. Environmental exposure to smoke plays a critical role in the etiology and development of asthma [5]. Passive smoking is associated with asthma directly through respiratory irritation and decreased pulmonary function [6-7] and these, in turn, lead to increasing asthma severity [8] and increased asthma prevalence [9]. Children who currently smoke or report exposure to secondhand smoke both at home and at school were found to be at increased risk of reporting active asthma symptoms, perform worse on lung function tests, and are more likely to develop chronic obstructive pulmonary disease as adults [6-7].

Prematurity/preterm birth and low birth weight (LBW) are other risk factors associated with childhood asthma. There is increasing evidence from meta-analyses suggesting that premature children are at a higher risk of asthma than term children independent of birth weight [10-11]; however, data from another meta-analysis found inconsistent findings across studies and suggest that there is no association between prematurity and asthma in about a third of the major studies analyzed [12]. LBW is also associated with preschool wheezing and asthma independent of prematurity [10], and a meta-analysis of 13 cohort studies suggests that LBW among children increases the risk of future asthma [13]. However, there are few studies that have observed the joint effects of birth weight and prematurity (BWP) on childhood asthma and asthma severity.

Additionally, little research has explored the joint moderating effect of BWP on the association of parental smoking and childhood asthma. Understanding the moderating role of BWP in asthmatic children living with smoking parents may increase the potential to intervene and improve health outcomes before they become clinically alarming. The aim of our study, therefore, was to examine the association between secondhand smoke exposure and childhood asthma and asthma severity among children aged six to 17. We hypothesized that BWP status would modify the association between parental smoking and childhood asthma.

## Materials and methods

We used the 2017 and 2018 data from the National Survey of Children’s Health (NSCH). NSCH is a cross-sectional study of non-institutionalized US children aged zero to 17 years and is sponsored by the Health Resources and Services Administration’s Maternal and Child Health Bureau since 2001 [14]. The NSCH surveys the physical and emotional health of children with an emphasis on factors related to child well-being, health care, family interactions, parental health, neighborhood characteristics, as well as school and after-school experiences. Information is collected from parents or guardians who know about the child's health. The 2017 and 2018 NSCH were administered online and by mail. Randomly selected addresses from households across the US were mailed instructions to access the survey online; some addresses also received a paper version of the screening questionnaire. The response rate for the 2017 and 2018 surveys were 37% and 43%, respectively [15].

Our dataset consisted of 30,530 and 21,599 children sampled from every state in the U.S. in 2017 and 2018, respectively. We excluded children zero to five years because the primary symptoms of asthma may be caused by other conditions, and respiratory diagnostic tests cannot be used easily or accurately in this age group [16]. NSCH received approval from the National Center for Health Statistics Research Ethics Review Board, and all participants provided informed consent. This study is exempt from full institutional review board review because the NSCH is a public use dataset. A detailed description of the data, sampling method, and other analytical guidelines are available elsewhere [17].

Assessments

A single item was used to ascertain the history of childhood asthma. Respondents were asked, “Has a doctor or other health professional ever told you that your child has asthma?” The child was considered to have asthma if the parent’s response was affirmative to this item. Asthma severity was assessed by inquiring, “How do you rate the severity of this child’s medical condition?” Responses were categorized as: "Does not currently have condition," "Current condition, rated mild," and "Current condition, rated moderate/severe." Self-reported physician diagnoses are reliable and valid measures of asthma status in both children and adults [18].

Smoking was assessed by two variables: parental smoking and secondhand smoke exposure. Parental smoking was ascertained by the questionnaire item “Do you smoke or use tobacco?” A “yes” response refers to any of the child’s parents or caregivers or both reporting a history of smoking or tobacco use. A “no” response refers to none of the child’s parents reporting smoking or tobacco use. Secondhand smoke exposure was assessed by the item “Does anyone smoke inside or outside the household?” and this question indicates if the child is living with any smoker (parents included). The responses were categorized as "No one smokes in the household," "Someone smokes, not inside the house" (outdoor smoking), and "Someone smokes inside the house" (indoor smoking).

The questionnaire item “Was this child born more than 3 weeks before his or her due date?” was used to assess prematurity status. A “yes” response was considered preterm birth. Birth weight was assessed with the survey question “How much did he or she weigh when born?” and categorized as low birth weight if birthweight was <2500 grams or normal weight if birthweight was ≥ 2500 grams. Based on these categories, a composite variable that combined both preterm status and birth weight was created as follows: “Normal birth weight and not premature” (NBW +NP), “low birth weight and not premature” (LBW+NP), “normal birth weight and premature” (NBW+P), and “low birth weight and premature” (LBW+P).

Other covariates are age (6-11 years and 12-17 years), sex (male or female), race/ethnicity (Hispanic, White non-Hispanic, Black non-Hispanic, multiracial/other races), socioeconomic status variables like the educational status of an adult in the household (“high school or lower” or “some college degree or higher”) and family structure (“two-parent family” or “no parent and single household”). Pesticide use in the home (“yes” vs “no”) and presence of mold in the home (“yes” vs “no”). This information was collected at the time of the survey.

Statistical analysis

The NSCH uses a stratified, multistage, complex survey design to enhance the representativeness of the U.S. children population. Analytical guidelines using complex analytical procedures suggested by the NSCH were followed. Appropriate weighting was applied to account for the sampling strata, cluster, and primary sampling unit. To account for this complex study design, we conducted a descriptive analysis of our study population. The Rao-Chi-Square test was used to conduct bivariate analysis between independent variables and the dependent variable (ever had asthma). Covariates were initially selected after a prior literature review of the risk factors associated with asthma. These explanatory variables were then statistically selected through both forward and backward selection. All explanatory variables met the SL/SE=0.10 criteria and were included in the model. Missing values were handled using multiple imputations. There was no difference between estimates of pooled 50 multiple-imputed datasets and estimates from the listwise deletion dataset. Therefore, we have reported the results of the listwise estimates.

Because the history of asthma was measured dichotomously, we estimated the unadjusted and adjusted binary logistic regression model (Model 1). Interaction between parental smoking and the composite BWP variable was statistically significant and was estimated in Model 2. Asthma severity had three levels (no current asthma, mild asthma, and moderate/severe), and we estimated the unadjusted and adjusted multinomial logistic regression models. We tabulated the results of logistic regression models as odds ratios (ORs) and 95% confidence intervals (CIs) for all independent variables for unadjusted, adjusted (Model 1), interaction model (Model 2), and the multinomial regression model. The variables adjusted in both the binary logistic model and the multinomial logistic model were age, sex, race, educational status of the parent in the household, family structure, presence of mold in the household, and pesticide use in the home. All percentages were reported as weighted percentages. The statistical hypothesis was tested using p < 0.05 as the level of significance. Data were analyzed using SAS 9.4 statistical software (SAS Institute, Cary, North Carolina).

## Results

Approximately 15.1% of our sample of 36,954 children aged six years and above reported ever having asthma (Table 1). The prevalence of smoking among parents was 15.4%. Twelve-point nine percent (12.9%) of children lived with an outdoor smoker while 2.4% of children had an indoor smoker. The sample had slightly higher males (51.1%) than females. Most of the participants were non-Hispanic white (50.2%), followed by Hispanic (25.6 %), non-Hispanic black (14.0%), and other races (10.1%). About one-third of parents (30.4%) had some college education or higher. Most children (72.8%) came from a two-parent household. Ten-point four percent (10.4%) of children had mold in their homes while about half of the children came from homes that used pesticides (50.7%). The percentage of children who reported LBW+NP, NBW+P, and LBW+P were 3.6%, 5.8%, and 5.9%, respectively.

**Table 1:** Descriptive statistics of the study sample (N = 36,954), children aged 6-17, National Survey of Children’s Health (NSCH), 2017-2018 Abbreviations: NBW, Normal birth weight; NP, Not premature; LBW, Low birth weight; P, Premature ^_a_^ Unweighted count ^b^ p values were determined by the χ2 test on weighted data.

		Non-Asthma (n=31378)		Asthma (n=5576)		
	Total sample, weighted (%)	Weighted freq (N^a^)	Weighted %	Weighted freq (N^a^)	Weighted%	p value ^b^
Age of Child						
6-11 years	49.9	21205294 (13694)	86.7	3249198 (2044)	13.3	<0.001
12-17 years	50.1	20557148 (17684)	83.9	3972948 (3532)	16.1	
Sex						
Female	48.9	20906444 (15420)	87.2	3070865 (2341)	12.8	<0.001
Male	51.1	20953787 (15958)	83.5	4151280 (3235)	16.5	
Race						
White non-Hispanic	50.2	21506759 (22051)	87.3	3139694 (3548)	12.7	<0.001
Hispanic	25.6	10736775 (3635)	85.5	1827689 (688)	14.5	
Black non-Hispanic	14.0	5441851 (1851)	78.9	1453887 (585)	21.1	
Multiracial/Others	10.1	4174846 (3841)	83.9	800875 (755)	16.1	
Parental smoking						
No	84.6	35042053 (26372)	86.1	5656688 (4500)	13.9	<0.001
Yes	15.4	6062817 (4549)	81.6	1363441 (18.4)	18.4	
Secondhand Smoking						
None	84.7	35042053 (26372)	86.1	5656688 (4500)	13.9	<0.001
Outdoor smoking	12.9	5075019 (3891)	81.7	6721905 (810)	18.3	
Indoor smoking	2.4	920268 (613)	80.5	1643783 (177)	19.5	
Composite Indicator						
NBW + NP	84.6	33406473 (25760)	86.0	5422826 (4305)	14.0	<0.001
LBW+ NP	3.6	1455151 (848)	87.2	213462 (138)	12.8	
NBW + P	5.8	2128902 (1626)	79.8	538852 (402)	20.2	
LBW+P	5.9	2126642 (1553)	78	599410 (429)	22.0	
Parent education						
Some college or higher	30.4	12746093 (5057)	85.5	2155079 (998)	14.5	0.72
High school or lower	69.6	29114139 (26321)	85.2	50567066 (4578)	14.8	
Family structure						
Two family structure	72.8	29995380 (23795)	86.1	4841710 (3898)	13.9	0.003
Single/no family structure	27.2	10876253 (7063)	83.5	2148094 (1578)	16.5	
Mold in home						
No	89.6	37002348 (28031)	85.8	6105913 (4798)	14.2	<0.001
Yes	10.4	4069659 (2855)	81.0	951942 (691)	9.0	
Home pesticide use						
No	49.3	19226657 (16078)	85.7	3218181 (2718)	14.3	0.61
Yes	50.7	19672080 (13569)	85.2	3405575 (2535)	14.8	

Parental smoking and secondhand smoke were associated with asthma in children aged six to 17 years in the unadjusted model (Table 2). In the unadjusted model, smoking parents were 1.39 times more likely to have children with asthma as compared to non-smoking parents (OR, 1.39; 95% CI, 1.19-1.64; p < .001). More so, children living with an outdoor smoker were 1.39 times more likely to report asthma as compared to children living with a non-smoker (OR, 1.39; 95% CI, 1.16-1.66; p < .001) while children living with an indoor smoker were 1.50 times more likely to report a child with asthma compared to children living with a non-smoker (OR, 1.50; 95% CI, 1.08-2.09; p =0.02) in the unadjusted model.

**Table 2:** Multivariate logistic regression of predictors against asthma among children aged 6-17, National Survey of Children’s Health (NSCH), 2017-2018 Abbreviations: NBW, Normal birth weight; NP, Not premature; LBW, Low birth weight; P, Premature ^a ^Model adjusted for age, sex, race/ethnicity, household smoking, parent education, family structure, mold in the home, home pesticide use, composite indicator ^b^ Interaction model: adjusted for age, sex, race/ethnicity, parental smoking, parent education, family structure, mold in home, home pesticide use, composite indicator

	Unadjusted Model		Model 1^a^		Model 2^a^	
Covariates	OR (95% CI)	p-value	OR (95% CI)	p-value	OR (95% CI)	p-value
Age						
6 -11 years	1 (Reference)		1 (Reference)		1 (Reference)	
12-17 years	1.26(1.11-1.43)	<0.001	1.34(1.17-1.53)	<0.001	1.34(1.18-1.54)	< .0001>
Sex						
Females	1 (Reference)		1 (Reference)		1 (Reference)	
Males	1.35(1.19-1.53)	<0.001	1.28(1.13-1.46)	<0.001	1.28(1.13-1.46)	<0.001
Race						
White non-Hispanic	1 (Reference)		1 (Reference)		1 (Reference)	
Hispanic	1.17(0.97-1.40)	0.08	1.18(0.96-1.45)	0.12	1.18(0.96-1.45)	0.11
Black non-Hispanic	1.83(1.55-2.16)	0.003	1.89(1.54-2.30)	<0.001	1.89(1.55-2.30)	<0.001
Multiracial/Others	1.31(1.11-1.56)	<0.001	1.38(1.14-1.68)	<0.001	1.39(1.14-1.68)	<0.001
Parental smoking						
No	1 (Reference)					
Yes	1.39(1.19-1.64)	<0.001				
Secondhand smoking						
None	1 (Reference)		1 (Reference)			
Outdoor smoking	1.39(1.16-1.66)	<0.001	1.27(1.06-1.52)	0.009		
Indoor smoking	1.50(1.08-2.09)	0.02	1.46(1.01-2.11)	0.04		
Parent education						
Some college	1 (Reference)		1 (Reference)		1 (Reference)	
Some high school	1.03 (0.88-1.20)	0.35	1.19 (0.991-1.43)	0.06	1.19 (0.99-1.43)	0.06
Family structure						
Two family structure	1 (Reference)		1 (Reference)		1 (Reference)	
Single/no family structure	1.22(1.07-1.40)	<0.001	1.10(0.91-1.23)	0.47	1.07(0.92-1.25)	0.40
Mold in home						
No	1 (Reference)		1 (Reference)			
Yes	1.42(1.17-1.72)	<0.001	1.37(1.14-1.66)	<0.001	1.38(1.14-1.66)	<0.001
Home pesticide use						
No	1 (Reference)		1 (Reference)		1 (Reference)	
Yes	1.03(0.91-1.18)	0.44	0.94(0.82-1.07)	0.35	0.94 (0.82-1.07)	0.39
Composite Indicator						
NBW + NP	1 (Reference)		1 (Reference)			
LBW+ NP	0.91(0.63-1.30)	0.62	0.91(0.62-1.32)	0.60		
NBW + P	1.56(1.21-2.01)	<0.001	1.63(1.37-2.09)	<0.001		
LBW+P	1.74(1.41-2.14)	<0.001	1.78(1.44-2.21)	<0.001		
Parental smoking*Composite indicator						
NBW+NP (Smoking “yes” vs “No”)					1.31(1.09-1.57)	0.003
LBW+ NP (Smoking “yes” vs “No”)					0.50(0.24-1.08)	0.08
NBW + P (Smoking “yes” vs “No”)					2.15(1.20-3.86)	0.01
LBW+P (Smoking “yes” vs “No”)					0.93(0.51-1.69)	0.80

The odds of asthma remained significant among children living with outdoor smokers (AOR, 1.27; 95% CI, 1.06-1.52; p=0.009) and indoor smokers (AOR, 1.46; 95% CI, 1.01-2.11; p =0.04) in Model 1 (Table 2) after controlling for the effect of the covariates in the model. Children aged 12-17 years were more likely to have asthma as compared to six to 11-year-old (AOR, 1.34; 95% CI, 1.17-1.53; p < .0001). Asthma was associated with mold in the home (AOR, 1.37; 95% CI, 1.14-1.66; p < .001), males (AOR, 1.28; 95% CI, 1.13-1.46; p < .001), and black race compared to white race (AOR, 1.87; 95% CI, 1.54-2.30; p < .001). Compared with NBW+NP children, asthma was associated with NBW+P (AOR, 1.63; 95% CI, 1.37-2.09; p < .001) and LBW+P (AOR, 1.78; 95% CI, 1.44-2.21; p < .001).

There was a statistically significant interaction between parental smoking and the BWP indicator in model 3 (Table 2). Normal weight children who were premature (NW+P) had the highest odds ratio (AOR, 2.15; 95% CI, 1.2-3.86) followed by NBW+NP children (AOR, 1.31; 95% CI, 1.09-1.17) after controlling for the covariates in the model.

In the adjusted multinomial logistic regression of predictors against asthma severity (Table 3), black non-Hispanic race was associated with increased odds of mild (AOR, 1.71; 95% CI, 1.36-2.30) and moderate/severe asthma (AOR, 2.94; 95% CI, 1.99-4.33). Having mold in the home increased the odds of mild asthma (AOR, 1.48; 95% CI, 1.15-1.91) and moderate/severe asthma (AOR, 1.72; 95% CI, 1.17-2.54). The risk of mild asthma was higher in NBW+P children (AOR, 1.88; 95% CI, 1.32-2.68). Mild and moderate/severe asthma odds were higher in LBW+P children (AOR, 1.90; 95% CI, 1.40-2.56 and AOR, 1.81; 95% CI, 1.16-2.84, respectively).

**Table 3:** Multinomial logistic regression of predictors against asthma severity among children aged 6-17, National Survey of Children’s Health (NSCH), 2017-2018 Abbreviations: NBW, Normal birth weight; NP, Not premature; LBW, Low Birth Weight; P, Premature ^a^. reference category are children with no current asthma

	Adjusted Model - Mild asthma ^a^		Adjusted Model - Moderate/ Severe asthma ^a^	
Covariates	OR (95% CI)	P value	OR (95% CI)	P value
Age				
6 -11 years	1 (Reference)		1 (Reference)	
12-17 years	1.19(0.99-1.44)	0.06	1.06(0.78-1.44)	0.71
Sex				
Females	1 (Reference)		1 (Reference)	
Males	1.07(0.89-1.29)	0.50	1.24(0.93-1.64)	0.14
Race				
White non-Hispanic	1 (Reference)		1 (Reference)	
Hispanic	1.01(0.76-1.35)	0.95	1.25(0.74-2.12)	0.41
Black non-Hispanic	1.76(1.35-2.29)	<0.001	2.94(1.99-4.33)	<0.001
Multiracial/Others	1.25(0.94-1.67)	0.13	1.16(0.78-1.73)	0.45
Secondhand smoking				
None	1 (Reference)		1 (Reference)	
Outdoor smoking	1.25(0.96-1.63)	0.10	1.37(0.98-1.91)	0.07
Indoor smoking	1.56(0.95-2.58)	0.08	1.44(0.74-2.78)	0.28
Parent education				
Some college and Higher	1 (Reference)		1 (Reference)	
High school and lower	1.19(0.90-1.57)	0.21	0.84(0.58-1.22)	0.36
Family structure				
Two family structure	1 (Reference)		1 (Reference)	
Single/no family structure	1.08(0.87-1.34)	0.49	1.27(0.91-1.77)	0.16
Mold in home				
No	1 (Reference)		1 (Reference)	
Yes	1.48(1.15-1.91)	0.002	1.72(1.17-2.54)	0.006
Home pesticide use				
No	1 (Reference)		1 (Reference)	
Yes	0.84(0.70-1.01)	0.06	1.01(0.74-1.36)	0.97
Composite Indicator				
NBW + NP	1 (Reference)		1 (Reference)	
LBW+ NP	1.04(0.55-1.98)	0.91	0.69(0.40-1.18)	0.17
NBW + P	1.88(1.32-2.68)	< .001>	1.30(0.82-2.10)	0.27
LBW+P	1.90(1.40-2.56)	< .001>	1.81(1.16-2.84)	0.001

## Discussion

Our study demonstrated a significant association between passive smoking and asthma in a nationally representative sample of US children aged six to 17. In the unadjusted analysis, parents who reported smoking were 39% more likely to report childhood asthma as compared to non-smoking parents. Secondhand smoke exposure was associated with increased odds of asthma in a dose-response manner in the unadjusted model (i.e., the odds ratio for asthma were 39% higher if a child who lived with an outdoor smoker as compared to a non-smoker and 50% higher if a child lived with an indoor smoker as compared to a non-smoker). After adjusting for several socioeconomic, demographic, and confounding factors, the association between secondhand smoke and asthma remained significant. Our study corroborates similar studies that found an increased report of asthma symptoms in children who reported passive smoking exposure both at home and in school [6-7].

We also found an association between the BWP composite indicators - NBW+P and LBW+P - and asthma in the unadjusted and adjusted model (Model 1). Our findings agree with several meta-analyses that have documented the relationship between low birth weight, prematurity, and asthma [10-11,13]. Considering the potentially deleterious joint effect of BWP on asthma, we further analyzed the moderating effect of this indicator on the association between parental smoking and asthma (Model 2). The interaction between parental smoking and BWP indicator variable was significant, indicating a modifying effect of BWP on the association between parental smoking and asthma. Specifically, the association between parental smoking and childhood asthma was high among NBW+NP and highest in NBW+P. This association was not significant in the LBW+P group. A reason for this result could be that the sample size of children who were LBW was not large enough to drive the interaction in this sub-group. The pathophysiologic mechanism for the moderating effect of prematurity and low birth weight on the relationship between smoking and asthma is still speculated. However, it is known that smoking impacts the respiratory epithelium through inhibition of the normal proliferation of fibroblasts, which are essential for lung repair and maintenance [19]. Smoking can also lead to airway hyperactivity, which heralds the development of asthma [20]. In addition, premature infants are at increased risk of respiratory symptoms, partially reversible airflow obstruction, and abnormal imaging in childhood and in young adulthood compared with term infants [21]. It has also been proposed that increasing lung function decline in preterm children especially if a child is exposed to noxious substances like cigarette smoke can cause chronic respiratory illness like asthma [21]. However, longitudinal studies to examine if the effects of prematurity and birth weight status are additive or multiplicative with smoking across the life course of asthma are warranted.

Our study utilized the NSCH database, which is a large and comprehensive survey of children’s health in the United States. The 2017 and 2018 NSCH studies covered over 50,000 children aged zero to 17 years, representative of the diversity of the U.S. Therefore, results from this dataset are reflective of the current state of children’s health in the U.S. Our sensitivity analysis, after accounting for missing values through multiple imputation showed that there was no difference between the listwise deleted dataset and the imputed dataset. Hence, the large size of the sample used in this analysis allowed for a precise estimate of the association between household smoking exposure and asthma in children, as well as the moderating effect of the BWP composite indicator on parent smoking and asthma.

We found increased odds of asthma to be higher in males as compared to females. Our results concur with prior studies that showed that male children had higher odds of physician-diagnosed asthma as compared to female children with asthma prevalence, later becoming greater in females after puberty and in adult life [22]. The etiology of these early asthma risks in males compared to females are unclear while increasing sex hormones have been attributed to the later asthma risk in women [23].

Another important finding of our study is that children who reported mold in their home were more likely to report asthma than those without mold. The association between mold and asthma severity was also statistically significant in children who reported mild asthma and moderate/severe asthma in a dose-response pattern. The association between mold and higher risk for asthma was shown in previous studies and a systematic review and meta-analysis in children concluded, for the first time, that there was sufficient evidence of a causal relationship between exposure to indoor molds and the development and exacerbation of asthma [24].

In this study, the Black race was strongly associated with childhood asthma and asthma severity in both unadjusted and adjusted logistic and adjusted multinomial models. Black non-Hispanics were three times more likely to report moderate/severe asthma as compared to White non-Hispanics. This finding supports previous findings indicating that non-Hispanic Black heads of household had higher odds of having a child diagnosed with asthma in the home as compared with non-Hispanic White heads, and the race-asthma association was decreased but not eliminated after controlling for homeownership and financial hardship [25].

Our study has several strengths. Our research included a large, population-based, representative sample of young children in the assessment of the association between childhood asthma, parent smoking, and important predictors thereby allowing broad generalization of our study results. Additionally, the NSCH data set also provides information on a large sample of the general population, rather than on a specific patient group, and thereby provides information on the degree of disease burden at this level. Statistical adjustments for differences in several health and lifestyle covariates helped provide a more detailed assessment of the strength of the association.

The limitations of our study include the use of parental self-reports for exposure and outcome ascertainment. Reporting bias may exist as parents may not be adequately trained to recognize the symptoms of asthma or consult healthcare providers early enough. Asthma severity was also ascertained through the parental report and the hierarchical ranking of the asthma severity was not in keeping with the known clinical classification of asthma severity [26]. The cross-sectional study design does not permit an assessment of the direction of the observed association and, therefore, causality cannot be inferred. The cross-sectional nature of this study also limited the scope of the secondary data analysis, as some putative risk factors were not included in the survey. There may also be a possibility of residual confounding due to the unavailability of confounders like body mass index (BMI) status, breastfeeding status, history of allergy and spirometry testing in the age groups, assessment of allergy and asthma in parents, ascertainment of bronchitis in children and physical activity status in the age group analyzed. Moreover, our study excluded children under the age of five and, therefore, we cannot generalize our study results to this population. Notwithstanding these limitations, this nationally representative sample provides some insight into the moderating role of BWP on the relationship between parental smoking and asthma among children in the United States.

## Conclusions

In conclusion, children aged six to 17 who live with a smoking parent or in a smoking household are at higher risk of asthma, and the risk is different by birth weight/preterm status. The association between smoking and asthma has public health implications: asthma primary and secondary preventions in children should screen for birth weight and prematurity status, as well as for smoke exposure from parents/guidance and in households.
